# Liver Metastatic Breast Cancer: Epidemiology, Dietary Interventions, and Related Metabolism

**DOI:** 10.3390/nu14122376

**Published:** 2022-06-08

**Authors:** Qianying Zuo, Nicole Hwajin Park, Jenna Kathryn Lee, Zeynep Madak Erdogan

**Affiliations:** 1 Department of Food Science and Human Nutrition, University of Illinois at Urbana-Champaign, Urbana, IL 61801, USA; qzuo2@illinois.edu (Q.Z.); nhpark2@illinois.edu (N.H.P.); 2 Department of Neuroscience, Northwestern University, Evanston, IL 60208, USA; jennalee0206@gmail.com; 3 Division of Nutritional Sciences, University of Illinois at Urbana-Champaign, Urbana, IL 61801, USA; 4 Cancer Center at Illinois, University of Illinois at Urbana-Champaign, Urbana, IL 61801, USA; 5 Beckman Institute for Advanced Science and Technology, University of Illinois at Urbana-Champaign, Urbana, IL 61801, USA; 6 Carl R. Woese Institute of Genomic Biology, University of Illinois at Urbana-Champaign, Urbana, IL 61801, USA; 7 Department of Biomedical and Translational Sciences, Carle-Illinois College of Medicine, University of Illinois at Urbana-Champaign, Urbana, IL 61801, USA

**Keywords:** breast cancer liver metastasis, western diet, fasting-mimicking diet

## Abstract

The median overall survival of patients with metastatic breast cancer is only 2–3 years, and for patients with untreated liver metastasis, it is as short as 4–8 months. Improving the survival of women with breast cancer requires more effective anti-cancer strategies, especially for metastatic disease. Nutrients can influence tumor microenvironments, and cancer metabolism can be manipulated via a dietary modification to enhance anti-cancer strategies. Yet, there are no standard evidence-based recommendations for diet therapies before or during cancer treatment, and few studies provide definitive data that certain diets can mediate tumor progression or therapeutic effectiveness in human cancer. This review focuses on metastatic breast cancer, in particular liver metastatic forms, and recent studies on the impact of diets on disease progression and treatment.

## 1. Introduction

Data from the American Cancer Society estimate that there will be 1.9 million new cancer cases diagnosed and 608,570 cancer deaths in the US in 2021 [1]. For women in the US, breast cancer is the most common cancer (30% of all new cases), with an estimated 281,550 newly diagnosed cases and 43,600 deaths in 2021 [1]. In 2018, an estimated 3.7 million women were living with breast cancer in the US [2]. Furthermore, global breast cancer mortality is increasing substantially, especially in developing regions such as Latin America and the Caribbean, rising by an estimated 7 million deaths every five years [3]. These trends demonstrate a need for continued efforts to abate a serious public health concern.

One emerging approach to intervene on breast cancer outcomes is the use of targeted dietary interventions. Indeed, accumulating data indicate that practical clinical dietary interventions, such as the ketogenic diet, can improve the efficacy of anticancer therapy [4]. Thus, dietary approaches hold potential to enhance therapeutic effectiveness and improve overall survival in breast cancer patients, thereby offering new promise for clinical practice that can change outcomes for a substantial number of patients worldwide.

Here, we review studies demonstrating how diet impacts disease progression and treatment in metastatic breast cancer, particularly metastases to liver.

## 2. Breast Cancer Metastasis

Approximately 63% of breast cancer patients are diagnosed with local-stage breast cancer, 27% with regional-stage disease, and 6% with distant (metastatic) disease [1]. In the US, an estimated >168,000 women were living with metastatic breast cancer in 2020 [5]. Although metastatic disease accounts for a small percentage of breast cancer cases, metastatic tumors are responsible for more than 90% of all cancer-related deaths [6]. Indeed, among breast cancer cases, the five-year survival rate for those with localized disease is more than 90%, but for those with metastases, the rate falls to just 28% [7]. Furthermore, the median survival of patients with metastatic disease at the time of diagnosis is approximately 18–24 months, and roughly 13% will survive 10 years [8]. About one-third of women diagnosed early with non-metastatic breast cancer will ultimately develop metastatic disease [9], which tends to develop resistance to therapies [10]. These phenomena underscore the increasing importance of developing therapies to prevent and treat metastatic disease and thus improve the overall survival of women with breast cancer [6].

The sites of distant metastasis among stage IV breast cancer patients include bone (68.8%), lung (16.0%), liver (13.3%), and brain (1.9%) [11]. Based on limited therapy options and dire disease outcomes for patients with liver metastasis, we will focus on liver metastatic ER^+^ breast cancer in this review. Important data on the impact of location of metastases on patient survival come from the National Cancer Institute’s Surveillance, Epidemiology, and End Results (SEER), a network of tumor registries that include about 30% of the US population and harboring data from 1975 to 2017 [9]. Of the 2.4 million cancer patients within this database, 5.14% present with synchronous liver metastases (LM) [9,12]. Half of all breast cancer patients develop LM, which often carries poor survival [13]—as low as 4–8 months if the disease is left untreated [14]. Surprisingly, metastatic breast cancer in the liver is observed more frequently in younger women (occurring in 34.2% of all patients < 50 years) than older women (occurring in 8.9% of all patients ≥ 50 years) [15,16]. In addition, patients with hormone receptor (HR)+/HER2+ breast cancer with LM have a longer median survival than patients with HR+/HER2- and triple-negative breast cancer due to the introduction of HER2-targeted therapy [17,18]. Thus, liver metastatic disease represents an important subgroup of breast cancer diagnoses that warrants focused efforts to improve outcomes.

## 3. Breast Cancer Liver Metastasis Diagnosis, Therapies, and Potential Treatments

Breast cancer LM may at first be asymptomatic, but possible symptoms include fatigue and weakness, pain or discomfort in the mid-section, weight loss or poor appetite, swelling in the legs, fever, and/or a yellow tint to the skin or whites of the eyes [19]. It is often identified by liver function tests that detect liver disease or damage [20]. Diagnosis may also be facilitated through imaging (MRI (magnetic resonance imaging), CT (computed tomography), PET (positron emission tomography), and PET/CT) or biopsy [21].

Most patients with breast cancer LM are treated with either systemic medications or local treatment [22]. Chemotherapy, hormonal therapies, or targeted therapies are common systemic treatments [23]. Chemotherapy involves the use of anti-cancer drugs to destroy or damage cancer cells [24]. Hormonal therapies use drugs such as tamoxifen, aromatase inhibitors, and fulvestrant to target estrogen and help shrink or slow the growth of HR+ metastatic breast cancer [25,26,27]. Targeted therapies exploit specific characteristics of cancer cells to treat metastatic disease. Some common targeted therapeutics are everolimus, bevacizumab+paclitaxel, palbociclib, and ribociclib [28,29,30,31,32,33]. Table 1 describes more current options such as potential oral selective estrogen receptor degraders or other pathway inhibitors. Local treatments for breast cancer LM include surgery, radiation therapy, and local chemotherapy. Surgery is most often used when the liver is the only site of metastasis and the symptoms are severe. Radiation therapies such as stereotactic body radiation therapy and Y-90 (Yttrium 90) radioembolization deliver or target radiation therapy directly to tumors in the liver [34,35].

Endocrine therapies reduce breast cancer mortality and relieve symptoms, but some persistent tumor cells frequently develop resistance in the metastatic and adjuvant setting [36,37,38]. Liver metastatic estrogen receptor α (ERα)-positive breast cancer is currently incurable [39]. Some potential small molecule therapies show good tumor responses in metastatic breast cancers. Axl kinase is associated with aggressive migratory behavior in tumors in a mouse model, and a combination of R428, a selective small molecule Axl inhibitor, with cisplatin positively reinforces both agents to block liver micro-metastases [40]. VERU-111 acts by depolymerizing microtubules, often leading to cell apoptosis due to the inability to complete mitosis, and is highly effective especially against fibrous tumors and metastases [41]. Recent evidence indicates that ErSO, a small molecule activator of a stress response mechanism that stimulates the anticipatory unfolded protein response (a-UPR), can eradicate most lung, bone, and liver metastases in orthotopic cell line xenograft and patient-derived xenograft (PDX) mouse models [39].

**Table 1:** Selected Oral Selective Estrogen Receptor Degraders or Other Inhibitors in Clinical Investigation.

Therapy	Administration	Target	Combination	Status	Year
Everolimus [42]	Oral	mTOR	Not noted	FDA approved	2020
Alpelisib [28,42]	Oral	PI3K-alpha	Combination with fulvestrantor letrozole	FDA approved	2020
Elacestrant [43]	Oral	Estrogen receptor	Low-fat diet combination	Phase Ib	2020
Giredestrant [44]	Oral	Estrogen receptor	Not noted	Phase III	2021
AZD9833 [45]	Oral	Estrogen receptor	Not noted	Phase I	2020

## 4. Link between Diets and Metastatic Breast Cancer

Dietary factors account for about 30% of cancer cases; thus, diet is one of the most modifiable causes of cancer [46]. High consumption of red meat, animal fats, and refined carbohydrates is associated with increased risk and severity of diseases such as breast cancer [47,48,49,50,51]. Furthermore, postmenopausal women have an increased risk of developing obesity-related breast cancer due to Western diets that promote weight gain, fat redistribution, dyslipidemia, hypertension, and insulin resistance, all of which are important in the recognition of metabolic syndrome [52,53,54]. For overweight or obese women, postmenopausal estrogen receptor-positive (ER+) and progesterone receptor-positive (HR+) breast cancer risks are about 1.5–2 times that of women with normal body weights [55,56]. This could be due to higher levels of estrogen produced by extra fat tissues in postmenopausal women and/or other mechanisms such as elevated levels of insulin [57,58]. Many studies have shown that weight gain also increases the risk of breast cancer in postmenopausal women compared with normal-weight women [59,60,61,62,63].

### 4.1. Western Diet

The Western diet is rich in fat and sugar, involving a high intake of saturated fats and sucrose and a low intake of fiber [64]. It plays a role in inflammatory disease and negatively affects both the immune system and gut microbiota [65]. Western diets are strongly associated with obesity and other metabolic effects such as weight gain and are often blamed for “the obesity epidemic”, as well as rising incidences of type 1 and type 2 diabetes [66].

**Figure 1:**
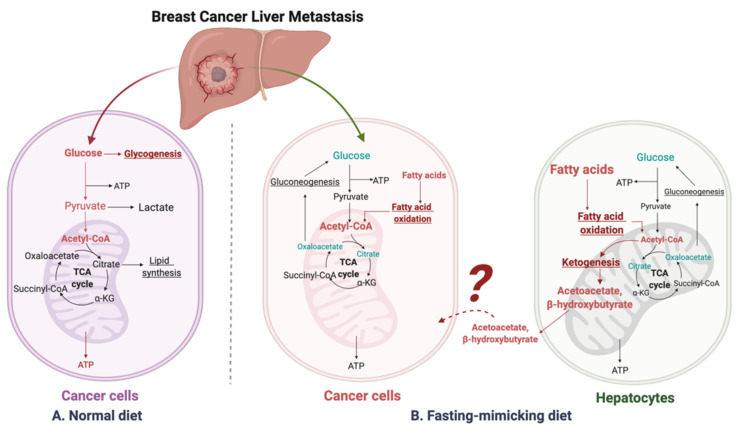
Suggested liver metastatic cancer metabolism in normal diet or fasting-mimicking diet. In the presence of glucose, the breast cancer cells metastasized to the liver mainly go through glycolysis to produce pyruvate, which is converted to acetyl-CoA via oxidative decarboxylation. Acetyl-CoA enters the tricarboxylic acid cycle (TCA cycle) and generates adenosine-3-phosphate (ATP) for cell survival and proliferation. Excessive glucose can be stored as glycogen for further usage. When glucose is limited (under a fasting-mimicking diet), the cancer cells switch to acquire ATP by fatty acid oxidation. The fatty acid oxidation is also highly activated in peripheral hepatocytes, where abundant acetyl-CoA feeds into ketogenesis and produces a large amount of acetoacetate and β-hydroxybutyrate. The liver does not use ketone bodies for energy because it lacks the necessary enzyme thiophorase (beta ketoacyl-CoA transferase). The unclear part is how these released ketone bodies function on breast cancer cells.

Glucose is central to the Western diet, so it is important to understand how cancer cells behave on a primary glucose energy source. (Figure 1). The Western diet directly promotes tumor cell proliferation via mechanisms involving the insulin/insulin-like growth factor 1 (IGF1)/phosphoinositide 3-kinase (PI3K) signaling pathway [67]. During regular cell function, glucose stimulates pancreatic β cells to release insulin, allowing glucose to enter cells to be used as a fuel source [68]. The high intake of carbohydrates and glucose stimulates the pancreas to increasingly secrete more insulin, which promotes the interaction of growth hormone receptors and growth hormones [69]. This elevates the levels of free IGF-1 released from the liver, which are associated with cell growth and proliferation and can harm cancer patients [70]. IGF-1 stimulates phosphorylation and activation of the serine/threonine kinase Akt via PI3K signaling. Akt then activates mammalian target of rapamycin (mTOR) and induces aerobic glycolysis by c-Myc and hypoxia-inducible factor (HIF)-1α. Insulin also stimulates interleukin 6 (IL-6) and tumor necrosis factor α (TNF-α) release [71,72,73].

There is evidence that deregulated glucose signaling influences cancer. Overexpression of glucose transporters 1 and 3 occurs in many aggressive tumors, and this correlates with elevated glucose uptake [74,75]. Furthermore, reducing glucose concentrations significantly decreases the proliferation of MCF-7 and T47D breast cancer cells and MCF-10A breast epithelial cells [76]. High glucose (25 mM) levels significantly abrogate the therapeutic effects of metformin on triple-negative breast cancer cell proliferation, death, and cell cycle arrest and contributed to metastatic progression and the development of resistance to chemotherapy/radiotherapy [77]. Additionally, mice with breast cancer liver metastasis fed sugar-rich diets had high metastatic burden, while mice fed high-fat/low-sugar diets had low tumor burden despite obesity [78]. In other mice studies, hepatocellular carcinoma tumor burden positively correlated with hepatic fat accumulation and insulin and liver IL-6 levels and inversely correlated with adiponectin levels [79,80]. These results indicate that dietary sugar intake may stimulate liver tumor growth.

Analysis of metabolic pathway components in mice indicates that reduced extracellular glucose can stimulate coactivator-associated arginine methyltransferase 1 (CARM1) to methylate GAPDH at R234, decreasing its likelihood of associating with its coenzyme NAD+. This inhibits the enzymatic activity of GAPDH and represses glycolysis to delay liver cancer cell growth, as cancer cells depend on glycolysis for proliferation [81]. Numerous other studies found that cancer cells become more dependent on blood glucose due to the demand for rapid cell growth, while others indicate that glucose may directly or indirectly affect tumor cell proliferation [77,82,83,84]. Notably, there is a higher incidence of breast cancer among diabetic and obese populations, contributing to the theory that a low-carbohydrate diet may limit or prevent tumor growth [85]. Emerging evidence supports a role of dietary interventions to counteract this putative effect.

### 4.2. Fasting-Mimicking Diet

The fasting-mimicking diet is low in calories from sugars, and protein but high in unsaturated fats. This diet is widely studied in relation to disease prevention and treatment, and although it has low levels of toxicity, it may have limits in terms of diet adherence and preexisting nutritional deficiencies [86]. Unlike the Western diet, low-carbohydrate diets slow cancer by inhibiting insulin/IGF and downstream intracellular signaling pathways, specifically PI3K/Akt/mTOR. Indeed, fasting prevents a Warburg shift, curbs glycolysis, and impedes AKT/mTOR signaling to improve the therapeutic response of Sorafenib-resistant hepatocellular carcinoma via p53-dependent metabolic synergism [82]. Increased AMP-activated protein kinase (AMPK) levels, stimulated by adenosine monophosphate, inhibit aerobic glycolysis and suppress proliferation, migration, and invasion of tumor cells [87].

Fluorodeoxyglucose-positron emission tomography (FDG-PET) demonstrates that most human cancer cells have a higher demand for glucose than surrounding non-cancer cells [88,89]. Metastatic cancer cells typically resemble cells of the primary cancer, but they can also be influenced by the milieu of the organs they colonize. Metabolic reprogramming happens after cells metastasize and colonize the liver. Liver cancer cells, like most other cancers, perform metabolic rewiring to increase their energy metabolism, becoming dependent on glucose or fructose as energy sources to fuel high rates of glycolysis or fructolysis, respectively [75,90,91,92], resulting in the use of the pentose phosphate pathway and glycolysis to generate NADPH and pyruvate [93,94,95] (Figure 1, left panel). For instance, dietary fructose provides fuel for major pathways of central carbon metabolism during tumor cell proliferation by activating the enzyme aldolase B (ALDOB) or its upstream regulator GATA6 in colon cancer liver metastasis [92]. Cancer cells become dependent on adenosine triphosphate (ATP) produced by the less efficient process of glycolysis [96]. Furthermore, tumor cells have more mitochondrial DNA mutations than normal cells, producing an increased number of reactive oxygen species (ROS) during respiration [97]. These cells are less capable of producing NADPH because gluconeogenesis cannot be performed to form the glucose-6-phosphate (G-6-P) necessary to enter the pentose phosphate pathway [98].

When glucose is limited, the body produces an alternative form of energy for its cells. Under the fasting-mimicking diet, cancer cells are forced to use mitochondrial oxidative metabolism, which causes metabolic oxidative stress as well as the production of ketones for energy instead (Figure 1, right panel). Ketone bodies produced by the liver benefit normal cells but not cancer cells [99,100]. As part of the Warburg effect, lactate is produced in excess, which compensates for dysfunctional mitochondrial oxidative phosphorylation [74,101,102]. High-fat, low-carbohydrate diets such as the fasting-mimicking diet are notable due to their ability to restrict the availability of glucose and limit a Warburg-type metabolism [103], further supporting that these diets have the potential to prevent or reverse tumor growth.

These series of events mean tumor cells are dependent on glucose, and this dependency can be exploited with the fasting-mimicking diet to selectively starve tumors by providing fat and protein that the tumor cells cannot use [104]. Several animal studies of various cancer types showed that the fasting-mimicking diet effectively limits tumor growth by itself or in combination with other therapies without causing the rebound hyperglycemia and hyperinsulinemia [100,101,105,106,107,108,109,110,111,112,113]. Metastatic TNBC patients with lower glycemia survive longer compared with those with higher glycemia. FMD reduces TNBC cancer stem cells (CSCs) and delays tumor progression [114]. Synergistic anti-neoplastic effects of the metformin/hypoglycemia combination were regulated by PP2A-GSK3β-MCL-1 axis, leading to a decline in the pro-survival protein MCL-1 and reduction in tumor growth in in vitro and in vivo metastatic melanomas models [115]. Furthermore, the very low carbohydrate diet reduces tumor incidence in a spontaneous mouse model of breast cancer [101]. In the metastatic 4T1 mouse mammary tumor model, combining a low-carbohydrate/high-protein diet and a cyclooxygenase-2 inhibitor significantly lowers the levels of breast cancer lung metastasis [108]. A low-carbohydrate diet also suppresses prostate cancer tumor growth in mice compared to a Western diet, which increases serum insulin, blood glucose, and tumor tissue insulin receptor levels [107]. Finally, a fasting-mimicking diet synergizes with classical chemotherapy to treat metastatic murine pancreas cancer in preclinical models by decreasing tumor glucose and glycolytic intermediates, increasing β-hydroxybutyrate and boosting reactive oxygen species [112].

### 4.3. β-Hydroxybutyrate Paradox

On a high-fat/low-carbohydrate diet, a process called ketogenesis produces ketone bodies in the liver [103,116] through the production of acetyl-coenzyme A (CoA) [117]. When the supply of liver carbohydrates is low, acetyl-CoA is broken down to acetoacetate (AcAc) and then further reduced to β-hydroxybutyrate (βHB), one of the most abundant and principle ketone bodies [116,118]. Though βHB is derived in the liver from the β-oxidation of free fatty acids (FFAs), the liver does not use ketone bodies for energy because it lacks the necessary enzyme thiophorase (beta ketoacyl-CoA transferase) [119] (Figure 1, right panel). In most humans, the plasma βHB concentration is typically at least 2 mM on a low-carbohydrate/high-fat diet [120]. βHB, a major metabolite of the fasting-mimicking diet, helps regulate many metabolic diseases due to its ability to control signaling events [116], e.g., the PI3K/Akt/mTOR pathways [110,121,122]. A fasting-mimicking diet enhances the anti-cancer efficacy of the endocrine therapeutics including tamoxifen and fulvestrant and delays endocrine resistance by lowering circulating IGF1, insulin, and leptin and by inhibiting AKT–mTOR signaling via upregulation of EGR1 and PTEN in mouse models of hormone-receptor-positive breast cancer [110]. Vernieri, Claudio, et al. reported the FMD first-in-human clinical trial (NCT03340935) in patients with different tumor types (including 56 breast cancer patients, 26 luminal, 19 TNBC, and 11 HER+) and treated with concomitant antitumor therapies. The FMD favorably modulates systemic and intratumor immunity and activates several antitumor immune programs by significantly reducing plasma glucose concentration, serum insulin, and serum IGF1 [123]. Some of its other key molecular targets include the NLRP3 inflammasome, RNA-binding proteins, and G protein-coupled receptors [124].

βHB has anti-inflammatory properties [122,125] and is characterized as an epigenetic modifier that produces anti-cancer effects by modifying chromatin and inhibiting histone deacetylases [126,127]. However, some studies link βHB to tumor progression, metastasis, and clinical failure [128,129,130,131]. These inverse effects gave rise to the “β-hydroxybutyrate paradox” theory [131].

Metastatic cancer models indicate that exogenous ketones have direct cytotoxic effects on the viability and survival of tumors [87]. For example, βHB effectively inhibits S2-013 cells, a cloned subline derived from a liver metastasis, with consequent metabolic reprogramming, of a human pancreatic tumor line (SUIT-2) [132]. Maldonado et al. demonstrated that low-carbohydrate diet-induced glucose deprivation is a potential strategy to enhance breast cancer treatment—very high βHB levels (25 mM) do not stimulate breast cancer cell proliferation, suggesting that breast cancer cells cannot use βHB as fuel to proliferate [76]. In an in vivo study of mice implanted with VM-M3 tumors, dietary ketone supplementation (either 1,3-butanediol or a ketone ester, which are metabolized to the ketone bodies βHB and acetoacetate) prolonged survival and reduced tumor burden in mice with metastatic cancer. In addition, supplementation lowers blood glucose, elevates blood ketones, and decreases overall body weight [87]. In another study, βHB enhances cisplatin-induced apoptosis via the histone deacetylase (HDAC)3/6 inhibition/survival axis in hepatocellular carcinoma [133]. Furthermore, clinical trials at the University of Würzburg tested low-carbohydrate/high-fat diets in 16 patients with advanced/metastatic solid malignant tumors and found that 3 months of ketogenic diet therapy resulted in a stable physical condition, lower body mass index, a somewhat better quality of life, and/or slowed tumor growth [134].

The β-hydroxybutyrate paradox indicates that βHB’s effect on cancer growth depends on the tumor’s energetic phenotype. Cells with an “oxidative phosphorylation phenotype” use βHB as an additional energy source whenever it is available, while cells with a “glycolytic, Warburg-like phenotype” cannot metabolize βHB, causing it to accumulate within the cell and inhibit tumor growth through cell signaling and epigenetic mechanisms [131,135]. An in vitro study determined that βHB can change the energetic phenotype of breast cancer cells but not their glucose consumption and production of lactate [135]. Furthermore, in a spontaneous mouse mammary tumor model, βHB at low concentration (<1 mM) increased tumor growth by acting as an oxidative energy source rather than as an epigenetic factor [131].

In addition to the β-hydroxybutyrate paradox, there is a paradox surrounding the common ketone body butyrate. The butyrate paradox suggests that, like βHB, butyrate can mediate histone acetylation and inhibit cell proliferation in cells following the Warburg effect and preferentially using glucose [131,136,137,138]. However, in cancer cells that do not follow the Warburg effect and oxidize butyrate as fuel, butyrate fails to reach inhibitory concentrations and can stimulate tumor growth [131]. An in vitro study showed that sodium butyrate (NaBu), an HDAC inhibitor, inhibits breast cancer cell growth in a time- and dose-dependent manner. This possible anti-cancer effect is due to NaBu eliciting apoptosis through elevated levels of ROS, increased caspase activity, and reduced mitochondrial membrane potential [139]. Luo et al. also demonstrated that NaBu induces autophagy in colorectal cancer cells through phosphorylated liver kinase B1 (LKB1)/AMPK signaling [138].

Overall, there are still many controversial opinions about high-fat/low-glucose diets, and the scientific community has not reached a consensus on its benefits or detriments. However, a fasting-mimicking diet can reduce the toxic effects of chemotherapy and enhance therapeutic efficacy beyond chemotherapy alone [140,141]. When used alongside chemotherapy, the fasting-mimicking diet delays breast cancer and melanoma progression in mice by reducing HO-1 to sensitize tumors to chemotherapy [142]. When applied to individuals with HER2-early breast cancer during chemotherapy, the fasting-mimicking diet increased tumor cell death and significantly slowed chemotherapy-induced DNA damage in T-lymphocytes [140,141]. Both animal studies and clinical trials are ongoing to better understand the mechanism of the fasting-mimicking diet and βHB-altered tumor microenvironments in specific cancer types.

## 5. Conclusions and Future Perspective

Globally, breast cancer is the most prevalent cancer in women, and metastatic disease is highly predictive of shortened survival. Improving the general survival of women with metastatic breast cancer requires more effective anti-cancer strategies in combination with current therapies or medications. Nutrition plays an important role before/during/after cancer treatment. While dietary modifications mostly have positive effects in the context of specific cancers, it is critical to optimize future investigations on metabolic therapies to understand how dietary factors and pharmacotherapies influence carcinogenesis based on tumor- and patient-related characteristics. To further improve the effects of the fasting-mimicking diet on the quality of life or cancer progression, more clinical studies are needed, as the safety and efficacy of the fasting-mimicking diet strongly depends on the tumor variety and its genotype. Understanding diet-associated molecular mechanisms involved in therapy resistance, especially the “β-hydroxybutyrate paradox theory”, will help reduce mortality and morbidity associated with metastatic breast cancer and prolong the quality-adjusted life expectancy of cancer survivors.

## References

[1] Tarver T. (2021). Breast Cancer Facts & Figures 2021.

[2] National Cancer Institute (2020). Cancer Stat Facts: Female Breast Cancer.

[3] Azamjah N., Soltan-Zadeh Y., Zayeri F. (2019). Global Trend of Breast Cancer Mortality Rate: A 25-Year Study. Asian Pac. J. Cancer Prev..

[4] Lévesque S., Pol J.G., Ferrere G., Galluzzi L., Zitvogel L., Kroemer G. (2019). Trial watch: Dietary interventions for cancer therapy. OncoImmunology.

[5] Mariotto A.B., Etzioni R., Hurlbert M., Penberthy L., Mayer M. (2017). Estimation of the Number of Women Living with Metastatic Breast Cancer in the United States. Cancer Epidemiol. Prev. Biomark..

[6] Gupta G.P., Massagué J. (2006). Cancer metastasis: Building a framework. Cell.

[7] Siegel R.L., Miller K.D., Fuchs H.E., Jemal A. (2021). Cancer statistics, 2021. CA Cancer J. Clin..

[8] Eng L.G., Dawood S., Sopik V., Haaland B., Tan P.S., Bhoo-Pathy N., Warner E., Iqbal J., Narod S.A., Dent R. (2016). Ten-year survival in women with primary stage IV breast cancer. Breast Cancer Res. Treat..

[9] Howlader N., Noone A.M., Krapcho M., Miller D., Brest A., Yu M., Ruhl J., Tatalovich Z., Mariotto A., Lewis D.R. (2020). SEER Cancer Statistics Review, 1975–2017.

[10] Soni D.A., Ren Z., Hameed O., Chanda D., Morgan C.J., Siegal G.P., Wei S. (2015). Breast Cancer Subtypes Predispose the Site of Distant Metastases. Am. J. Clin. Pathol..

[11] Gong Y., Liu Y.-R., Ji P., Hu X., Shao Z.-M. (2017). Impact of molecular subtypes on metastatic breast cancer patients: A SEER population-based study. Sci. Rep..

[12] Horn S.R., Stoltzfus K.C., Lehrer E.J., Dawson L.A., Tchelebi L., Gusani N.J., Sharma N.K., Chen H., Trifiletti D.M., Zaorsky N.G. (2020). Epidemiology of liver metastases. Cancer Epidemiol..

[13] Rashid N.S., Grible J.M., Clevenger C.V., Harrell J.C. (2021). Breast cancer liver metastasis: Current and future treatment approaches. Clin. Exp. Metastasis.

[14] Adam R., Aloia T., Krissat J., Bralet M.P., Paule B., Giacchetti S., Delvart V., Azoulay D., Bishmuth H., Castaing D. (2006). Is liver resection justified for patients with hepatic metastases from breast cancer?. Ann. Surg..

[15] De Ridder J., De Wilt J.H.W., Simmer F., Overbeek L., Lemmens V., Nagtegaal I. (2016). Incidence and origin of histologically confirmed liver metastases: An explorative case-study of 23,154 patients. Oncotarget.

[16] Cummings M.C., Simpson P.T., Reid L.E., Jayanthan J., Skerman J., Song S., Reed A.E.M., Kutasovic J.R., Morey A.L., Marquart L. (2014). Metastatic progression of breast cancer: Insights from 50 years of autopsies. J. Pathol..

[17] Ji L., Cheng L., Zhu X., Gao Y., Fan L., Wang Z. (2021). Risk and prognostic factors of breast cancer with liver metastases. BMC Cancer.

[18] Xie J.Z., Xu A. (2019). Population-Based Study on Liver Metastases in Women with Newly Diagnosed Breast Cancer. Cancer Epidemiol Biomark. Prev..

[19] Diamond J.R., Finlayson C.A., Borges V.F. (2009). Hepatic complications of breast cancer. Lancet Oncol..

[20] Patanaphan V., Salazar O.M., Risco R. (1988). Breast cancer: Metastatic patterns and their prognosis. South. Med. J..

[21] Cao R., Wang L.-P. (2012). Serological Diagnosis of Liver Metastasis in Patients with Breast Cancer. Cancer Biol. Med..

[22] Bale R., Putzer D., Schullian P. (2019). Local Treatment of Breast Cancer Liver Metastasis. Cancers.

[23] Higgins M.J., Baselga J. (2011). Targeted therapies for breast cancer. J. Clin. Investig..

[24] Carrick S., Parker S., Thornton C.E., Ghersi D., Simes J., Wilcken N. (2009). Single agent versus combination chemotherapy for metastatic breast cancer. Cochrane Database Syst. Rev..

[25] Osborne C.K. (1998). Tamoxifen in the treatment of breast cancer. N. Engl. J. Med..

[26] Smith I.E., Dowsett M. (2003). Aromatase inhibitors in breast cancer. N. Engl. J. Med..

[27] Spoerke J.M., Gendreau S., Walter K., Qiu J., Wilson T.R., Savage H., Aimi J., Derynck M.K., Chen M., Chan I.T. (2016). Heterogeneity and clinical significance of ESR1 mutations in ER-positive metastatic breast cancer patients receiving fulvestrant. Nat. Commun..

[28] Mayer I.A., Abramson V.G., Formisano L., Balko J.M., Estrada M.V., Sanders M.E. (2017). A phase Ib study of alpelisib (BYL719), a PI3Kα-specific inhibitor, with letrozole in ER+/HER2− metastatic breast cancer. Clin. Cancer Res..

[29] Coleman R.L., Brady M.F., Herzog T.J., Sabbatini P., Armstrong D.K., Walker J.L., Kim B.G., Fujiwara K., Tewari K.S., O’Malley D.M. (2017). Bevacizumab and paclitaxel–carboplatin chemotherapy and secondary cytoreduction in recurrent, platinum-sensitive ovarian cancer (NRG Oncology/Gynecologic Oncology Group study GOG-0213): A multicentre, open-label, randomised, phase 3 trial. Lancet Oncol..

[30] Delaloge S., Pérol D., Courtinard C., Brain E., Asselain B., Bachelot T., Debled M., Dieras V., Campone M., Levy C. (2016). Paclitaxel plus bevacizumab or paclitaxel as first-line treatment for HER2-negative metastatic breast cancer in a multicenter national observational study. Ann. Oncol..

[31] Finn R.S., Martin M., Rugo H.S., Jones S., Im S.-A., Gelmon K., Harbeck N., Lipatov O.N., Walshe J.M., Moulder S. (2016). Palbociclib and Letrozole in Advanced Breast Cancer. N. Engl. J. Med..

[32] Turner N.C., Ro J., André F., Loi S., Verma S., Iwata H., Harbeck N., Loibl S., Bartlett C.H., Zhang K. (2015). Palbociclib in Hormone-Receptor–Positive Advanced Breast Cancer. N. Engl. J. Med..

[33] Hortobagyi G.N., Stemmer S.M., Burris H.A., Yap Y.-S., Sonke G.S., Paluch-Shimon S., Campone M., Blackwell K.L., André F., Winer E.P. (2016). Ribociclib as First-Line Therapy for HR-Positive, Advanced Breast Cancer. N. Engl. J. Med..

[34] Senkus E., Łacko A. (2017). Over-treatment in metastatic breast cancer. Breast.

[35] Cardoso F., Harbeck N., Fallowfield L., Kyriakides S., Senkus E. (2012). ESMO Guidelines Working Group. Locally recurrent or metastatic breast cancer: ESMO Clinical Practice Guidelines for diagnosis, treatment and follow-up. Ann. Oncol..

[36] Williams M., Lee L., Werfel T., Joly M.M.M., Hicks D.J., Rahman B., Elion D., McKernan C., Sanchez V., Estrada M.V. (2018). Intrinsic apoptotic pathway activation increases response to anti-estrogens in luminal breast cancers. Cell Death Dis..

[37] Richman J., Dowsett M. (2019). Beyond 5 years: Enduring risk of recurrence in oestrogen receptor-positive breast cancer. Nat. Rev. Clin. Oncol..

[38] Razavi P., Chang M.T., Xu G., Bandlamudi C., Ross D.S., Vasan N., Cai Y., Bielski C.M., Donoghue M.T.A., Jonsson P. (2018). The Genomic Landscape of Endocrine-Resistant Advanced Breast Cancers. Cancer Cell.

[39] Boudreau M.W., Duraki D., Wang L., Mao C., Kim J.E., Henn M.A., Tang B., Fanning S.W., Kiefer J., Tarasow T.M. (2021). A small-molecule activator of the unfolded protein response eradicates human breast tumors in mice. Sci. Transl. Med..

[40] Holland S.J., Pan A., Franci C., Hu Y., Chang B., Li W., Duan M., Torneros A., Yu J., Heckrodt T.J. (2010). R428, a Selective Small Molecule Inhibitor of Axl Kinase, Blocks Tumor Spread and Prolongs Survival in Models of Metastatic Breast Cancer. Cancer Res..

[41] Deng S., Krutilina R.I., Wang Q., Lin Z., Parke D.N., Playa H.C., Chen H., Miller D.D., Seagroves T.N., Li W. (2020). An Orally Available Tubulin Inhibitor, VERU-111, Suppresses Triple-Negative Breast Cancer Tumor Growth and Metastasis and Bypasses Taxane Resistance. Mol. Cancer Ther..

[42] Vernieri C., Corti F., Nichetti F., Ligorio F., Manglaviti S., Zattarin E., Rea C.G., Capri G., Bianchi G.V., De Braud F. (2020). Everolimus versus alpelisib in advanced hormone receptor-positive HER2-negative breast cancer: Targeting different nodes of the PI3K/AKT/mTORC1 pathway with different clinical implications. Breast Cancer Res..

[43] Jager A., De Vries E.G.E., Oordt C.W.M.-V.D.H.V., Neven P., Venema C.M., Glaudemans A.W.J.M., Wang Y., Bagley R.G., Conlan M.G., Aftimos P. (2020). A phase 1b study evaluating the effect of elacestrant treatment on estrogen receptor availability and estradiol binding to the estrogen receptor in metastatic breast cancer lesions using 18F-FES PET/CT imaging. Breast Cancer Res..

[44] Liang J., Zbieg J.R., Blake R.A., Chang J.H., Daly S., DiPasquale A.G., Friedman L.S., Gelzleichter T., Gill M., Giltnane J.M. (2021). GDC-9545 (Giredestrant): A Potent and Orally Bioavailable Selective Estrogen Receptor Antagonist and Degrader with an Exceptional Preclinical Profile for ER+ Breast Cancer. J. Med. Chem..

[45] Scott J.S., Moss T.A., Balazs A., Barlaam B., Breed J., Carbajo R.J., Chiarparin E., Davey P.R.J., Delpuech O., Fawell S. (2020). Discovery of AZD9833, a Potent and Orally Bioavailable Selective Estrogen Receptor Degrader and Antagonist. J. Med. Chem..

[46] Doll R., Peto R. (1981). The Causes of Cancer: Quantitative Estimates of Avoidable Risks of Cancer in the United States Today. J. Natl. Cancer Inst..

[47] Nicodemus K.K., Jacobsr D.R., Folsom A.R. (2001). Whole and refined grain intake and risk of incident postmenopausal breast cancer (United States). Cancer Causes Control.

[48] Dydjow-Bendek D., Zagozdzon P. (2020). Total Dietary Fats, Fatty Acids, and Omega-3/Omega-6 Ratio as Risk Factors of Breast Cancer in the Polish Population-a Case-Control Study. In Vivo.

[49] Tsilidis K.K., Travis R.C., Appleby P.N., Allen N.E., Lindström S., Albanes D., Ziegler R.G., McCullough M.L., Siddiq A., Barricarte A. (2013). Insulin-like growth factor pathway genes and blood concentrations, dietary protein and risk of prostate cancer in the NCI Breast and Prostate Cancer Cohort Consortium (BPC3). Int. J. Cancer.

[50] Williams C.M., Dickerson J.W. (1987). Dietary fat, hormones and breast cancer: The cell membrane as a possible site of interaction of these two risk factors. Eur. J. Surg. Oncol..

[51] Levi F., La Vecchia C., Gulie C., Negri E. (1993). Dietary Factors and Breast-Cancer Risk in Vaud, Switzerland. Nutr. Cancer Int. J..

[52] Mauvais-Jarvis F., Clegg D.J., Hevener A.L. (2013). The Role of Estrogens in Control of Energy Balance and Glucose Homeostasis. Endocr. Rev..

[53] Madak-Erdogan Z., Band S., Zhao Y.C., Smith B.P., Kulkoyluoglu-Cotul E., Zuo Q., Casiano A.S., Wrobel K., Rossi G., Smith R.L. (2019). Free fatty acids rewire cancer metabolism in obesity-associated breast cancer via estrogen receptor and mTOR signaling. Cancer Res..

[54] Zuo Q., Band S., Kesavadas M., Erdogan Z.M. (2021). Obesity and Postmenopausal Hormone Receptor-positive Breast Cancer: Epidemiology and Mechanisms. Endocrinology.

[55] Jiralerspong S., Goodwin P.J. (2016). Obesity and Breast Cancer Prognosis: Evidence, Challenges, and Opportunities. J. Clin. Oncol..

[56] Suzuki R., Orsini N., Saji S., Key T.J., Wolk A. (2009). Body weight and incidence of breast cancer defined by estrogen and progesterone receptor status-A meta-analysis. Int. J. Cancer.

[57] Iyengar N.M., Arthur R., Manson J.E., Chlebowski R.T., Kroenke C.H., Peterson L., Cheng T.D., Feliciano E., Lane D., Luo J. (2019). Association of Body Fat and Risk of Breast Cancer in Postmenopausal Women with Normal Body Mass Index A Secondary Analysis of a Randomized Clinical Trial and Observational Study. JAMA Oncol..

[58] Picon-Ruiz M., Morata-Tarifa C., Valle-Goffin J.J., Friedman E.R., Slingerland J.M. (2017). Obesity and adverse breast cancer risk and outcome: Mechanistic insights and strategies for intervention. CA Cancer J. Clin..

[59] Keum N., Greenwood D.C., Lee D.H., Kim R., Aune D., Ju W., Hu F.B., Giovannucci E.L. (2015). Adult Weight Gain and Adiposity-Related Cancers: A Dose-Response Meta-Analysis of Prospective Observational Studies. J. Natl. Cancer Inst..

[60] Renehan A.G., Tyson M., Egger M., Heller R.F., Zwahlen M. (2008). Body-mass index and incidence of cancer: A systematic review and meta-analysis of prospective observational studies. Lancet.

[61] Chan D.S.M., Vieira A.R., Aune D., Bandera E.V., Greenwood D.C., McTiernan A., Rosenblatt D.N., Thune I., Vieira R., Norat T. (2014). Body mass index and survival in women with breast cancer—systematic literature review and meta-analysis of 82 follow-up studies. Ann. Oncol..

[62] Gathirua-Mwangi W.G., Palmer J.R., Champion V., Castro-Webb N., Stokes A.C., Adams-Campbell L., Marley A.R., Forman M.R., Rosenberg L., Bertrand K.A. (2022). Maximum and Time-Dependent Body Mass Index and Breast Cancer Incidence Among Postmenopausal Women in the Black Women’s Health Study. Am. J. Epidemiol..

[63] Chauhan R., Trivedi V., Rani R., Singh U. (2020). A comparative analysis of body mass index with estrogen receptor, progesterone receptor and human epidermal growth factor receptor 2 status in pre- and postmenopausal breast cancer patients. J. Mid-Life Health.

[64] Statovci D., Aguilera M., Mac Sharry J., Melgar S. (2017). The Impact of Western Diet and Nutrients on the Microbiota and Immune Response at Mucosal Interfaces. Front. Immunol..

[65] Garcia-Montero C., Fraile-Martínez O., Gómez-Lahoz A.M., Pekarek L., Castellanos A.J., Noguerales-Fraguas F., Coca S., Guijarro L.G., García-Honduvilla N., Asúnsolo A. (2021). Nutritional Components in Western Diet Versus Mediterranean Diet at the Gut Microbiota-Immune System Interplay. Implications for Health and Disease. Nutrients.

[66] Zinöcker M.K., Lindseth I.A. (2018). The Western Diet–Microbiome-Host Interaction and Its Role in Metabolic Disease. Nutrients.

[67] Cully M., You H., Levine A.J., Mak T.W. (2006). Beyond PTEN mutations: The PI3K pathway as an integrator of multiple inputs during tumorigenesis. Nat. Rev. Cancer.

[68] Haythorne E., Rohm M., Van De Bunt M., Brereton M.F., Tarasov A.I., Blacker T.S., Sachse G., Dos Santos M.S., Exposito R.T., Davis S. (2019). Diabetes causes marked inhibition of mitochondrial metabolism in pancreatic β-cells. Nat. Commun..

[69] Newsholme P., Keane K.N., Carlessi R., Cruzat V. (2019). Oxidative stress pathways in pancreatic β-cells and insulin-sensitive cells and tissues: Importance to cell metabolism, function, and dysfunction. Am. J. Physiol. Cell Physiol..

[70] Klement R.J., Kammerer U. (2011). Is there a role for carbohydrate restriction in the treatment and prevention of cancer?. Nutr. Metab..

[71] LaPensee C.R., Hugo E.R., Ben-Jonathan N. (2008). Insulin Stimulates Interleukin-6 Expression and Release in LS14 Human Adipocytes through Multiple Signaling Pathways. Endocrinology.

[72] Makino T., Noguchi Y., Yoshikawa T., Doi C., Nomura K. (1998). Circulating interleukin 6 concentrations and insulin resistance in patients with cancer. Br. J. Surg..

[73] Mccall J.L., Tuckey J.A., Parry B.R. (1992). Serum Tumor-Necrosis-Factor-Alpha and Insulin Resistance in Gastrointestinal Cancer. Br. J. Surg..

[74] Tian M., Zhang H., Nakasone Y., Mogi K., Endo K. (2004). Expression of Glut-1 and Glut-3 in untreated oral squamous cell carcinoma compared with FDG accumulation in a PET study. Eur. J. Nucl. Med. Mol. Imaging.

[75] Zhang H.-L., Wang M.-D., Zhou X., Qin C.-J., Fu G.-B., Tang L., Wu H., Huang S., Zhao L.-H., Zeng M. (2017). Blocking preferential glucose uptake sensitizes liver tumor-initiating cells to glucose restriction and sorafenib treatment. Cancer Lett..

[76] Maldonado R., Talana C.A., Song C., Dixon A., Uehara K., Weichhaus M. (2021). β-hydroxybutyrate does not alter the effects of glucose deprivation on breast cancer cells. Oncol. Lett..

[77] Varghese S., Samuel S.M., Varghese E., Kubatka P., Büsselberg D. (2019). High Glucose Represses the Anti-Proliferative and Pro-Apoptotic Effect of Metformin in Triple Negative Breast Cancer Cells. Biomolecules.

[78] Zuo Q., Mogol A.N., Liu Y.-J., Casiano A.S., Chien C., Drnevich J., Imir O.B., Kulkoyluoglu-Cotul E., Park N.H., Shapiro D.J. (2022). Targeting metabolic adaptations in the breast cancer-liver metastatic niche using dietary approaches to improve endocrine therapy efficacy. Mol. Cancer Res..

[79] Healy M.E., Chow J.D., Byrne F.L., Breen D.S., Leitinger N., Li C., Lackner C., Caldwell S.H., Hoehn K.L. (2015). Dietary effects on liver tumor burden in mice treated with the hepatocellular carcinogen diethylnitrosamine. J. Hepatol..

[80] Bechmann L.P., Hannivoort R.A., Gerken G., Hotamisligil G.S., Trauner M., Canbay A. (2012). The interaction of hepatic lipid and glucose metabolism in liver diseases. J. Hepatol..

[81] Zhong X.-Y., Yuan X.-M., Xu Y.-Y., Yin M., Yan W.-W., Zou S.-W., Wei L.-M., Lu H.-J., Wang Y.-P., Lei Q.-Y. (2018). CARM1 Methylates GAPDH to Regulate Glucose Metabolism and Is Suppressed in Liver Cancer. Cell Rep..

[82] Krstic J., Reinisch I., Schindlmaier K., Galhuber M., Riahi Z., Berger N., Kupper N., Moyschewitz E., Auer M., Michenthaler H. (2022). Fasting improves therapeutic response in hepatocellular carcinoma through p53-dependent metabolic synergism. Sci. Adv..

[83] Wahdan-Alaswad R.S., Edgerton S.M., Salem H.S., Thor A.D. (2018). Metformin Targets Glucose Metabolism in Triple Negative Breast Cancer. J. Oncol. Transl. Res..

[84] Roy R., Hahm E.R., White A.G., Anderson C.J., Singh S.V. (2019). AKT-dependent sugar addiction by benzyl isothiocyanate in breast cancer cells. Mol. Carcinog..

[85] Gluschnaider U., Hertz R., Ohayon S., Smeir E., Smets M., Pikarsky E., Bar-Tana J. (2014). Long-Chain Fatty Acid Analogues Suppress Breast Tumorigenesis and Progression. Cancer Res..

[86] Wei M., Brandhorst S., Shelehchi M., Mirzaei H., Cheng C.W., Budniak J., Groshen S., Mack W.J., Guen E., Di Biase S. (2017). Fasting-mimicking diet and markers/risk factors for aging, diabetes, cancer, and cardiovascular disease. Sci. Transl. Med..

[87] Poff A., Ari C., Arnold P., Seyfried T., D’Agostino D. (2014). Ketone supplementation decreases tumor cell viability and prolongs survival of mice with metastatic cancer. Int. J. Cancer.

[88] Allen B.G., Bhatia S.K., Anderson C.M., Eichenberger-Gilmore J.M., Sibenaller Z.A., Mapuskar K.A., Schoenfeld J.D., Buatti J.M., Spitz D.R., Fath M.A. (2014). Ketogenic diets as an adjuvant cancer therapy: History and potential mechanism. Redox Biol..

[89] Rigo P., Paulus P., Kaschten B.J., Hustinx R., Bury T., Jerusalem G., Benoit T., Foidart-Willems J. (1996). Oncological applications of positron emission tomography with fluorine-18 fluorodeoxyglucose. Eur. J. Nucl. Med..

[90] Kaji K., Nishimura N., Seki K., Sato S., Saikawa S., Nakanishi K., Furukawa M., Kawaratani H., Kitade M., Moriya K. (2018). Sodium glucose cotransporter 2 inhibitor canagliflozin attenuates liver cancer cell growth and angiogenic activity by inhibiting glucose uptake. Int. J. Cancer.

[91] Zhang X., Qiao Y., Wu Q., Chen Y., Zou S., Liu X., Zhu G., Zhao Y., Chen Y., Yu Y. (2017). The essential role of YAP O-GlcNAcylation in high-glucose-stimulated liver tumorigenesis. Nat. Commun..

[92] Bu P., Chen K.-Y., Xiang K., Johnson C., Crown S.B., Rakhilin N., Ai Y., Wang L., Xi R., Astapova I. (2018). Aldolase B-Mediated Fructose Metabolism Drives Metabolic Reprogramming of Colon Cancer Liver Metastasis. Cell Metab..

[93] Warburg O. (1956). On the Origin of Cancer Cells. Science.

[94] Anykin-Burns N., Ahmad I.A., Zhu Y., Oberley L.W., Spitz D.R. (2009). Increased levels of superoxide and hydrogen peroxide mediate the differential susceptibility of cancer cells vs. Normal cells to glucose deprivation. Biochem. J..

[95] Boros L.G., Lee P.W.N., Brandesa J.L., Cascante M., Muscarellaa P., Schirmera W.J., Melvina W.S., Ellisona E.C. (1998). Nonoxidative pentose phosphate pathways and their direct role in ribose synthesis in tumors: Is cancer a disease of cellular glucose metabolism?. Med. Hypotheses.

[96] Weber D.D., Aminazdeh-Gohari S., Kofler B. (2018). Ketogenic diet in cancer therapy. Aging.

[97] Wallace D.C. (2012). Mitochondria and cancer. Nat. Rev. Cancer.

[98] Buettner G.R. (2011). Superoxide dismutase in redox biology: The roles of superoxide and hydrogen peroxide. Anti-Cancer Agents Med. Chem..

[99] Veech R.L. (2014). Ketone ester effects on metabolism and transcription. J. Lipid Res..

[100] Hopkins B.D., Pauli C., Du X., Wang D.G., Li X., Wu D., Amadiume S.C., Goncalves M.D., Hodakoski C., Lundquist M.R. (2018). Suppression of insulin feedback enhances the efficacy of PI3K inhibitors. Nature.

[101] Ho V.W., Leung K., Hsu A., Luk B., Lai J., Shen S.Y., Minchinton A.I., Waterhouse D., Bally M., Lin W. (2011). A Low Carbohydrate, High Protein Diet Slows Tumor Growth and Prevents Cancer Initiation. Cancer Res..

[102] Vander Heiden M.G., Cantley L.C., Thompson C.B. (2009). Understanding the Warburg Effect: The Metabolic Requirements of Cell Proliferation. Science.

[103] Byrne F.L., Hargett S.R., Lahiri S., Roy R.J., Berr S.S., Caldwell S.H., Hoehn K.L. (2018). Serial MRI Imaging Reveals Minimal Impact of Ketogenic Diet on Established Liver Tumor Growth. Cancers.

[104] Tan-Shalaby J. (2017). Ketogenic diets and cancer: Emerging evidence. Fed. Pract..

[105] Allen B.G., Bhatia S.K., Buatti J., Brandt K.E., Lindholm K.E., Button A., Szweda L.I., Smith B.J., Spitz D.R., Fath M.A. (2013). Ketogenic Diets Enhance Oxidative Stress and Radio-Chemo-Therapy Responses in Lung Cancer Xenografts. Clin. Cancer Res..

[106] Abdelwahab M.G., Fenton K.E., Preul M.C., Rho J.M., Lynch A., Stafford P., Scheck A.C. (2012). The Ketogenic Diet Is an Effective Adjuvant to Radiation Therapy for the Treatment of Malignant Glioma. PLoS ONE.

[107] Fokidis H.B., Chin M.Y., Ho V.W., Adomat H.H., Soma K.K., Fazli L., Nip K.M., Cox M., Krystal G., Zoubeidi A. (2015). A low carbohydrate, high protein diet suppresses intratumoral androgen synthesis and slows castration-resistant prostate tumor growth in mice. J. Steroid Biochem. Mol. Biol..

[108] Ho V.W., Hamilton M.J., Dang N.-H.T., Hsu B.E., Adomat H.H., Guns E.S., Weljie A., Samudio I., Bennewith K.L., Krystal G. (2014). A low carbohydrate, high protein diet combined with celecoxib markedly reduces metastasis. Carcinogenesis.

[109] Martuscello R.T., Vedam-Mai V., McCarthy D.J., Schmoll M.E., Jundi M.A., Louviere C.D., Griffith B.G., Skinner C.L., Suslov O., Deleyrolle L.P. (2016). A Supplemented High-Fat Low-Carbohydrate Diet for the Treatment of Glioblastoma. Clin. Cancer Res..

[110] Caffa I., Spagnolo V., Vernieri C., Valdemarin F., Becherini P., Wei M., Brandhorst S., Zucal C., Driehuis E., Ferrando L. (2020). Fasting-mimicking diet and hormone therapy induce breast cancer regression. Nature.

[111] Klement R.J., Champ C.E., Otto C., Kämmerer U. (2016). Anti-Tumor Effects of Ketogenic Diets in Mice: A Meta-Analysis. PLoS ONE.

[112] Yang L., TeSlaa T., Ng S., Nofal M., Wang L., Lan T., Zeng X., Cowan A., McBride M., Lu W. (2022). Ketogenic diet and chemotherapy combine to disrupt pancreatic cancer metabolism and growth. Med.

[113] Di Biase S., Shim H.S., Kim K.H., Vinciguerra M., Rappa F., Wei M., Brandhorst S., Cappello F., Mirzaei H., Lee C. (2017). Fasting regulates EGR1 and protects from glucose-and dexamethasone-dependent sensitization to chemotherapy. PLoS Biol..

[114] Salvadori G., Zanardi F., Iannelli F., Lobefaro R., Vernieri C., Longo V.D. (2021). Fasting-mimicking diet blocks triple-negative breast cancer and cancer stem cell escape. Cell Metab..

[115] Elgendy M., Cirò M., Hosseini A., Weiszmann J., Mazzarella L., Ferrari E., Cazzoli R., Curigliano G., DeCensi A., Bonanni B. (2019). Combination of Hypoglycemia and Metformin Impairs Tumor Metabolic Plasticity and Growth by Modulating the PP2A-GSK3β-MCL-1 Axis. Cancer Cell.

[116] Puchalska P., Crawford P.A. (2017). Multi-dimensional Roles of Ketone Bodies in Fuel Metabolism, Signaling, and Therapeutics. Cell Metab..

[117] Laffel L. (1999). Ketone bodies: A review of physiology, pathophysiology and application of monitoring to diabetes. Diabetes/Metab. Res. Rev..

[118] Newman J.C., Verdin E. (2017). β-Hydroxybutyrate: A Signaling Metabolite. Annu. Rev. Nutr..

[119] Dhillon K.K., Gupta S. (2022). Biochemistry, Ketogenesis. StatPearls.

[120] Kim D.Y., Rho J.M. (2008). The ketogenic diet and epilepsy. Curr. Opin. Clin. Nutr. Metab. Care.

[121] Duan Y., Zhang Y., Dong H., Wang Y., Zhang J. (2017). Effects of dietary poly-beta-hydroxybutyrate (PHB) on microbiota composition and the mTOR signaling pathway in the intestines of litopenaeus vannamei. J. Microbiol..

[122] Huang C., Wang P., Xu X., Zhang Y., Gong Y., Hu W., Gao M., Wu Y., Ling Y., Zhao X. (2018). The ketone body metabolite beta-hydroxybutyrate induces an antidepression-associated ramification of microglia via HDACs inhibition-triggered Akt-small RhoGTPase activation. Glia.

[123] Vernieri C., Fucà G., Ligorio F., Huber V., Vingiani A., Iannelli F., Raimondi A., Rinchai D., Frigè G., Belfiore A. (2022). Fasting-Mimicking Diet Is Safe and Reshapes Metabolism and Antitumor Immunity in Patients with Cancer. Cancer Discov..

[124] Han Y.-M., Ramprasath T., Zou M.-H. (2020). β-hydroxybutyrate and its metabolic effects on age-associated pathology. Exp. Mol. Med..

[125] Kim D.H., Park M.H., Ha S., Bang E.J., Lee Y., Lee A.K., Lee J., Yu B.P., Chung H.Y. (2019). Anti-inflammatory action of beta-hydroxybutyrate via modulation of PGC-1alpha and FoxO1, mimicking calorie restriction. Aging.

[126] Shimazu T., Hirschey M.D., Newman J., He W., Shirakawa K., Le Moan N., Grueter C.A., Lim H., Saunders L.R., Stevens R.D. (2013). Suppression of oxidative stress by beta-hydroxybutyrate, an endogenous histone deacetylase inhibitor. Science.

[127] Pant K., Peixoto E., Richard S., Gradilone S.A. (2020). Role of Histone Deacetylases in Carcinogenesis: Potential Role in Cholangiocarcinoma. Cells.

[128] Martinez-Outschoorn U.E., Prisco M., Ertel A., Tsirigos A., Lin Z., Pavlides S., Wang C., Flomenberg N., Knudsen E.S., Howell A. (2011). Ketones and lactate increase cancer cell “stemness,” driving recurrence, metastasis and poor clinical outcome in breast cancer: Achieving personalized medicine via Metabolo-Genomics. Cell Cycle.

[129] Bonuccelli G., Tsirigos A., Whitaker-Menezes D., Pavlides S., Pestell R.G., Chiavarina B., Frank P.G., Flomenberg N., Howell A., Martinez-Outschoorn U.E. (2010). Ketones and lactate “fuel” tumor growth and metastasis: Evidence that epithelial cancer cells use oxidative mitochondrial metabolism. Cell Cycle.

[130] Huang C., Chang P., Kuo W., Chen C., Jeng Y., Chang K., Shew J., Hu C., Lee W. (2017). Adipocytes promote malignant growth of breast tumours with monocarboxylate transporter 2 expression via beta-hydroxybutyrate. Nat. Commun..

[131] Rodrigues L.M., Uribe-Lewis S., Madhu B., Honess D.J., Stubbs M., Griffiths J.R. (2017). The action of β-hydroxybutyrate on the growth, metabolism and global histone H3 acetylation of spontaneous mouse mammary tumours: Evidence of a β-hydroxybutyrate paradox. Cancer Metab..

[132] Shukla S.K., Gebregiworgis T., Purohit V., Chaika N.V., Gunda V., Radhakrishnan P., Mehla K., Pipinos I.I., Powers R., Yu F. (2014). Metabolic reprogramming induced by ketone bodies diminishes pancreatic cancer cachexia. Cancer Metab..

[133] Mikami D., Kobayashi M., Uwada J., Yazawa T., Kamiyama K., Nishimori K., Nishikawa Y., Nishikawa S., Yokoi S., Taniguchi T. (2020). β-Hydroxybutyrate enhances the cytotoxic effect of cisplatin via the inhibition of HDAC/survivin axis in human hepatocellular carcinoma cells. J. Pharmacol. Sci..

[134] Schmidt M., Pfetzer N., Schwab M., Strauss I., Kämmerer U. (2011). Effects of a ketogenic diet on the quality of life in 16 patients with advanced cancer: A pilot trial. Nutr. Metab..

[135] Bartmann C., Raman S.R.J., Flöter J., Schulze A., Bahlke K., Willingstorfer J., Strunz M., Wöckel A., Klement R.J., Kapp M. (2018). Beta-hydroxybutyrate (3-OHB) can influence the energetic phenotype of breast cancer cells, but does not impact their proliferation and the response to chemotherapy or radiation. Cancer Metab..

[136] Donohoe D.R., Collins L.B., Wali A., Bigler R., Sun W., Bultman S.J. (2012). The Warburg Effect Dictates the Mechanism of Butyrate-Mediated Histone Acetylation and Cell Proliferation. Mol. Cell.

[137] Lupton J.R. (2004). Microbial Degradation Products Influence Colon Cancer Risk: The Butyrate Controversy. J. Nutr..

[138] Luo S., Li Z., Mao L., Chen S., Sun S. (2019). Sodium butyrate induces autophagy in colorectal cancer cells through LKB1/AMPK signaling. J. Physiol. Biochem..

[139] Salimi V., Shahsavari Z., Safizadeh B., Hosseini A., Khademian N., Tavakoli-Yaraki M. (2017). Sodium butyrate promotes apoptosis in breast cancer cells through reactive oxygen species (ROS) formation and mitochondrial impairment. Lipids Health Dis..

[140] Vernieri C., Ligorio F., Zattarin E., Rivoltini L., De Braud F. (2020). Fasting-mimicking diet plus chemotherapy in breast cancer treatment. Nat. Commun..

[141] De Groot S., Lugtenberg R.T., Cohen D., Welters M.J.P., Ehsan I., Vreeswijk M.P.G., Smit V.T.H.B.M., de Graaf H., Heijns J.B., Portielje J.E.A. (2020). Fasting mimicking diet as an adjunct to neoadjuvant chemotherapy for breast cancer in the multicentre randomized phase 2 DIRECT trial. Nat. Commun..

[142] Di Biase S., Lee C., Brandhorst S., Manes B., Buono R., Cheng C.-W., Cacciottolo M., Martin-Montalvo A., De Cabo R., Wei M. (2016). Fasting-Mimicking Diet Reduces HO-1 to Promote T Cell-Mediated Tumor Cytotoxicity. Cancer Cell.

